# A nomogram based on combining clinical features and contrast enhanced ultrasound is not able to identify Her-2 over-expressing cancer from other breast cancers

**DOI:** 10.3389/fonc.2023.1035645

**Published:** 2023-01-26

**Authors:** Zi-mei Lin, Ting-ting Wang, Jun-Yan Zhu, Yong-yuan Xu, Fen Chen, Pin-tong Huang

**Affiliations:** ^1^ Department of Ultrasound in Medicine, The Second Affiliated Hospital Zhejiang University School of Medicine, Hangzhou, China; ^2^ Department of Ultrasound, The First Affiliated Hospital of Zhejiang Chinese Medical University, Hangzhou, China

**Keywords:** microbubbles, ultrasonography, breast cancer, biomarkers, nomogram

## Abstract

**Objective:**

The aim of this study was to evaluate whether a predictive model based on a contrast enhanced ultrasound (CEUS)-based nomogram and clinical features (Clin) could differentiate Her-2-overexpressing breast cancers from other breast cancers.

**Methods:**

A total of 152 pathology-proven breast cancers including 55 Her-2-overexpressing cancers and 97 other cancers from two units that underwent preoperative CEUS examination, were included and divided into training (n = 102) and validation cohorts (n = 50). Multivariate regression analysis was utilized to identify independent indicators for developing predictive nomogram models. The area under the receiver operating characteristic (AUC) curve was also calculated to establish the diagnostic performance of different predictive models. The corresponding sensitivities and specificities of different models at the cutoff nomogram value were compared.

**Results:**

In the training cohort, 7 clinical features (menstruation, larger tumor size, higher CA153 level, BMI, diastolic pressure, heart rate and outer upper quarter (OUQ)) + enlargement in CEUS with *P* < 0.2 according to the univariate analysis were submitted to the multivariate analysis. By incorporating clinical information and enlargement on the CEUS pattern, independently significant indicators for Her-2-overexpression were used for further predictive modeling as follows: Model I, nomogram model based on clinical features (Clin); Model II, nomogram model combining enlargement (Clin + Enlargement); Model III, nomogram model based on typical clinical features combining enlargement (MC + BMI + diastolic pressure (DP) + outer upper quarter (OUQ) + Enlargement). Model II achieved an AUC value of 0.776 at nomogram cutoff score value of 190, which was higher than that of the other models in the training cohort without significant differences (all *P*>0.05). In the test cohort, the diagnostic efficiency of predictive model was poor (all AUC<0.6). In addition, the sensitivity and specificity were not significantly different between Models I and II (all *P*>0.05), in either the training or the test cohort. In addition, Clin exhibited an AUC similar to that of model III (*P*=0.12). Moreover, model III exhibited a higher sensitivity (70.0%) than the other models with similar AUC and specificity, only in the test cohort.

**Conclusion:**

The main finding of the study was that the predictive model based on a CEUS-based nomogram and clinical features could not differentiate Her-2-overexpressing breast cancers from other breast cancers.

## Introduction

Breast cancer is the most common cancer diagnosed and the fifth leading cause of cancer death among Chinese women (1). Recurrence and metastasis are the main causes of treatment failure and death in patients with breast cancer (2).

Numerous studies have concentrated on the molecular diagnosis and prognosis of breast cancer since the incidence rate and prognosis of breast diseases with different pathological types vary greatly (3–6). The human epidermal growth factor receptor 2 gene (Her-2) is a member of the HER family, which prevents apoptosis and promotes cell proliferation (7, 8). Breast cancers that overexpress Her-2 are aggressive, accounting for 25% of all breast cancer cases (1, 9). Patients with Her-2 positive breast cancer have a lower survival rate than those without Her-2 overexpression. Her-2 has been used as a predictive and prognostic biomarker for breast cancer (10–12). However, the results of immunohistochemistry are limited by tumor heterogeneity and volume. Many imaging techniques (e.g. ultrasound (US), mammography and magnetic resonance imaging (MRI)) can provide morphological information about breast tumors. BI-RADS-US is helpful for differentiating benign and malignant lesions. Mammography is the recommended screening test around the world. However, mammography has low sensitivity in patients with dense breasts, especially for Chinese women, which may cause delayed diagnosis and worse outcomes (13). Compared with MRI, US is a widely available, low-cost technique that is less time consuming. The American College of Radiology published Breast Imaging Reporting and Data System (BI-RADS) lexicon guidelines for breast cancer screening, to standardize image interpretation by radiologists and dictate management recommendations. Despite improved consistency, the interpretation of US features is operator-dependent and objective, which contributes to further interobserver variation (14). In addition, malignancies present overlapping US features between Her-2 overexpressing cancers and other malignancies (OMs) (15–17). Studies have evaluated the relationships between prognosis and preoperative demographic information and serum and cancer biomarkers, but they have reported contrasting findings (18, 19). Previous studies including our study found that the features on contrast enhanced ultrasound (CEUS) are significant tools for characterizing breast lesions (20–22). CEUS enables real-time scanning by injecting blood-pool agents and truly reflects the vascular condition within the tumor microenvironment with great convenience and cost-effectiveness. CEUS features [i.e., hyper-enhancement, sun-sign and enlargement] can be used as biosignatures for identifying aggressive biological behavior (20–22). However, the abovementioned studies were all carried out at a single center. Moreover, variable definitions of CEUS features in these studies inhibit their further clinical applications. To the best of our knowledge, studies have not yet evaluated the prognostic values of preoperative CEUS in estimating breast cancer classification. Hence, we aimed at to evaluate whether a predictive model based on a CEUS-based nomogram and clinical features (Clin) could differentiate Her-2-overexpressing breast cancers from OMs.

## Methods

### Patients

We retrospectively analyzed detailed clinical and pathological data from breast cancer patients. The requirement to obtain informed consent for study inclusion was waived. However, all patients undergoing CEUS, biopsy, or surgery signed informed consent forms for these examinations or procedures. All patients underwent a conventional US and CEUS before core biopsy and/or surgery. Patients with previously-diagnosed breast cancer or incomplete clinical information were excluded, and male patients were excluded as well. None of the patients had received preoperative chemotherapy or radiotherapy. Clinical information included several independent variables such as demographics (age, menstrual cycle [MC], family history of cancer, blood pressure, heartrate [HR], CA153, and body mass index [BMI]), histopathological features (histopathological type, pathologic stage of regional lymph node (pTN) stage, size of invasive component in millimeters, multifocality/multicentricity status, and lymph node status), and the expression of ER, PR, HER2, and Ki67. The ER, PR, HER2, Ki-67-labeling index and histological type were confirmed by surgery.

In total, 102 patients at the Second Affiliated Hospital Zhejiang University School of Medicine were included as the training set from January 2018 to June 2021. Another 50 patients from the Second Affiliated Hospital Zhejiang University School of Medicine and the First Affiliated Hospital of Zhejiang Chinese Medical University were prospectively included as the internal and external validation sets to validate the predictive model between July 2021 and July 2022.

### US

US images of breast masses were obtained using a Resona 7/7S/9 scanner (Mindray, China), and MyLab TM Twice (Esaote, Italy) equipped with a 3- to 11-MHz linear probe and a 4- to 13-MHz linear probe by one of seven senior radiologists with 5–15 years of experience in conventional US and at least 2 years of experience in CEUS of the breast. The nodule size was defined by the maximal diameter on US. The number and location of the masses were also recorded. If multiple masses were present, the most suspicious (the higher BI-RADS category) or the largest mass was targeted. The machine settings were adjusted to obtain optimal US images, and the images were stored for further analysis.

### CEUS and analysis

The same transducer as use with US equipped with contrast-specific, continuous-mode software was used for CEUS. Patients were instructed to breathe quietly during the entire process. A second-generation US contrast agent (sulfur hexafluoride, SonoVue; Bracco, Milan, Italy) was intravenously administered at a dose of 4.8 mL and was subsequently manually flushed with 5 mL of saline. Starting at the beginning of the saline injection, a 120-second-long clip was documented during the examination. We manually outlined the area most perfused within the mass as a selected ROI (≈5.0 mm^2^), from which the following mean perfusion parameters were extrapolated: The plane with the most abundant vessels was selected as the CEUS target area. For lesions with no blood detected on color Doppler flow imaging (CDFI), the section with the most irregular shape was chose instead. The plane with maximal diameter was chosen as the last choice when a mass with a regular shape and no blood was detected. Then, time–intensity curves (TICs) using the local density random walk wash-in, wash-out (LDRW-WIWO) method was acquired using built-in software. Finally, we obtained the following parameters: (1) the enhancement echogenicity (hetero-enhancement or homo-enhancement); (2) the enhancement intensity (hyper-enhancement, hypo-enhancement, or iso-enhancement); (3) the enhancement shape (regular or irregular); (4) the enhancement border (well-defined or ill-defined border); (5) the enhancement size (larger than vs. equal to the US size); and (6) the crab-like sign (present or absent, defined as the nourishing vessels around the tumor). The features of hyperenhancement, enlarged, and crab-like sign in the contrast mode are related to malignant lesions, according to the findings of our earlier study and other studies (18–20). If the CEUS result was positive, the original BI-RADS score remained unchanged. If the CEUS result was negative, the original BI-RADS score was downgraded one level (e.g., BI- RADS 4A was downgraded to BI-RADS 3).

US and CEUS images and clips were assessed by two senior radiologists in consensus (1:15 years of experience in breast US and 8 years of experience in CEUS; 2:6 years of experience in breast US and 5 years of experience in CEUS). All radiologists were blinded to the patients’ clinical data and pathology results.

### Histopathological analysis and scoring

The histopathology results after surgery were used as the final diagnosis of the masses. Histopathological specimen assessments were carried out on formalin-fixed paraffin-embedded tissue sections selected to include representative sections of carcinomas and adjacent normal breast tissue. Tumor cell staining was compared with that of the surrounding normal breast epithelium, which was used as the negative control. The slides were scored according to the percentage of positive cells vs. total cell number, regardless of staining intensity for non-standardized biomarkers. The immunostaining scores for ER, PR, and Ki67 and the algorithm for HER2 scoring were determined according to the ASCO and CAP guidelines (23, 24). Cell proliferation (Ki67) was assessed by nuclear staining in at least 500 tumor cells using a mouse monoclonal antibody, clone MIB1 (Dako Denmark A/S, Glostrup, Denmark) at a 1/100 dilution. By convention, we considered the expression level of Ki-67 to be low if the percentage of nuclear staining was <20%, intermediate if between 21% and 60%, and high if ≥60%. The tissue sections were examined by two pathologists with 10 and 15 years of experience in histopathology who were blinded to the clinical information.

### Statistical analysis

Continuous variables are expressed as the mean ± standard deviation while categorical variables are expressed as percentages according to normal distribution tests. Continuous data were compared by independent t tests while categorical data were compared using Chi-square tests or Fisher’s tests if necessary. In the training cohort, significant parameters between Her-2-overexpressing and Her-2-negative patients with *P*<0.2 were enrolled in the multivariate regression model by the stepwise forward selection method. Then, independently significant indicators for Her-2 overexpressing were used for further predictive model establishment as follows: Model I, nomogram model based on clinical features (Clin); Model II, nomogram model combining enlargement (Clin + Enlargement); Model III, nomogram model based on typical clinical features combining enlargement (MC + BMI + diastolic pressure (DP) + outer upper quarter (OUQ) + Enlargement). The diagnostic performances of the predictive models were tested in both the training and test cohorts. The area under the receiver operating characteristics curve (AUC) was established to indicate the diagnostic performance of different predictive models. Comparisons of AUC were determined using the Delong test, both in the training and test cohorts. The sensitivities, specificities, positive predictive values (PPV) and negative predictive values (NPV) were compared by the chi-square test. Inter observer agreement was calculated by the intraclass coefficient (ICC) model. Statistical analyses were performed by the SPSS 23.0 software package (Chicago, USA) and Medcalc software (Mariakerke, Belgium). *P*<0.05 was taken as the threshold for statistical significance.

## Results

### Baseline characteristics

The incidences of Her-2-overexpressing breast cancers were 34.3% and 40.0% in the training and test cohorts, respectively. As shown in **Supplemental Table 1**, baseline parameters did not significantly differ between the training and test cohorts.

In univariate analysis (**Table 1**), elevated BMI levels were found in the Her-2-overexpressing group (*P*<0.05) of the training population. In contrast, there were no CEUS features that were significantly associated with Her-2 overexpressing (all *P >*0.05). For the test cohort, elevated systolic pressure levels were more prevalent in the Her-2-overexpressing population (*P*<0.05). Moreover, no CEUS features that were significantly associated with Her-2 overexpressing (all *P >*0.05) (**Supplemental Table 2**).

**Table 1:** Univariate analysis of Clinical, US and CEUS features for predicting Her-2 overexpressing in training cohort. .

	Level	Overall	OMs	Her-2+	*P*
n		102	67	35	
age (mean (SD))		55.96 (12.57)	56.25 (12.62)	55.40 (12.63)	0.746
Menstruation(0=no;1=yes)	0	31 (30.4)	17 (25.4)	14 (40.0)	0.174
	1	71 (69.6)	50 (74.6)	21 (60.0)	
BMI (median [IQR])		22.70 [21.23, 24.99]	22.31 [20.59, 24.88]	24.14 [21.89, 25.04]	0.049
BMI≥25	0	78 (76.5)	52 (77.6)	26 (74.3)	0.807
	1	24 (23.5)	15 (22.4)	9 (25.7)	
Systolic (mean (SD))		123.65 (15.78)	122.61 (14.69)	125.64 (17.75)	0.36
Systolic≥140	0	89 (87.3)	60 (89.6)	29 (82.9)	0.361
	1	13 (12.7)	7 (10.4)	6 (17.1)	
Diastolic (mean (SD))		75.53 (9.98)	74.32 (9.80)	77.83 (10.07)	0.092
Diastolic≥90	0	94 (92.2)	63 (94.0)	31 (88.6)	0.441
	1	8 (7.8)	4 (6.0)	4 (11.4)	
Diastolic≥80	0	69 (67.6)	48 (71.6)	21 (60.0)	0.269
	1	33 (32.4)	19 (28.4)	14 (40.0)	
Heart rate (mean (SD))		81.24 (14.27)	82.58 (14.82)	78.67 (12.98)	0.19
Heartrate≥100	0	93 (91.2)	59 (88.1)	34 (97.1)	0.159
	1	9 (8.8)	8 (11.9)	1 (2.9)	
CA153 (median [IQR])		9.00 [6.40, 11.78]	8.30 [6.40, 11.05]	9.10 [7.35, 13.15]	0.184
CA153≥14	0	87 (85.3)	59 (88.1)	28 (80.0)	0.377
	1	15 (14.7)	8 (11.9)	7 (20.0)	
CA153≥20	0	96 (94.1)	65 (97.0)	31 (88.6)	0.177
	1	6 (5.9)	2 (3.0)	4 (11.4)	
CA153≥25	0	98 (96.1)	66 (98.5)	32 (91.4)	0.116
	1	4 (3.9)	1 (1.5)	3 (8.6)	
Lesion location(1=left;2=right)	1	57 (55.9)	37 (55.2)	20 (57.1)	1
	2	45 (44.1)	30 (44.8)	15 (42.9)	
o'clock (0=areola)	0	19 (18.6)	16 (23.9)	3 (8.6)	0.306
	1	6 (5.9)	4 (6.0)	2 (5.7)	
	2	16 (15.7)	13 (19.4)	3 (8.6)	
	3	7 (6.9)	3 (4.5)	4 (11.4)	
	4	6 (5.9)	4 (6.0)	2 (5.7)	
	5	3 (2.9)	2 (3.0)	1 (2.9)	
	7	7 (6.9)	4 (6.0)	3 (8.6)	
	8	4 (3.9)	2 (3.0)	2 (5.7)	
	9	4 (3.9)	2 (3.0)	2 (5.7)	
	10	15 (14.7)	10 (14.9)	5 (14.3)	
	11	4 (3.9)	2 (3.0)	2 (5.7)	
	12	11 (10.8)	3 (4.5)	6 (17.1)	
Areola	0	83 (81.4)	51 (76.1)	32 (91.4)	0.067
	1	19 (18.6)	16 (23.9)	3 (8.6)	
OUQ	0	57 (55.9)	42 (62.7)	15 (42.9)	0.062
	1	45 (44.1)	25 (37.3)	20 (57.1)	
Size (median [IQR])		1.54 [1.13, 2.06]	1.52 [1.14, 1.93]	1.64 [1.13, 2.17]	0.617
Size≥2	0	74 (72.5)	52 (77.6)	22 (62.9)	0.16
	1	28 (27.5)	15 (22.4)	13 (37.1)	
Size≥2.5	0	86 (84.3)	57 (85.1)	29 (82.9)	0.78
	1	16 (15.7)	10 (14.9)	6 (17.1)	
Size≥3	0	93 (91.2)	62 (92.5)	31 (88.6)	0.489
	1	9 (8.8)	5 (7.5)	4 (11.4)	
BIRADS category	3	37 (36.3)	24 (35.8)	13 (37.1)	0.695
	4A	29 (28.4)	20 (29.9)	9 (25.7)	
	4B	33 (32.4)	22 (32.8)	11 (31.4)	
	4C	3 (2.9)	1 (1.5)	2 (5.7)	
BIRADS 4B	0	73 (71.6)	47 (70.1)	26 (74.3)	0.818
	1	29 (28.4)	20 (29.9)	9 (25.7)	
BIRADS 4C	0	69 (67.6)	45 (67.2)	24 (68.6)	1
	1	33 (32.4)	22 (32.8)	11 (31.4)	
BIRADS 5	0	99 (97.1)	66 (98.5)	33 (94.3)	0.27
	1	3 (2.9)	1 (1.5)	2 (5.7)	
Elastography	2	4 (3.9)	4 (6.0)	0 (0.0)	0.285
	3	50 (49.0)	34 (50.7)	16 (45.7)	
	4	39 (38.2)	25 (37.3)	14 (40.0)	
	5	9 (8.8)	4 (6.0)	5 (14.3)	
E3	0	52 (51.0)	33 (49.3)	19 (54.3)	0.68
	1	50 (49.0)	34 (50.7)	16 (45.7)	
E4	0	63 (61.8)	42 (62.7)	21 (60.0)	0.832
	1	39 (38.2)	25 (37.3)	14 (40.0)	
E5	0	93 (91.2)	63 (94.0)	30 (85.7)	0.268
	1	9 (8.8)	4 (6.0)	5 (14.3)	
CEUS BIRADS category	3	2 (2.0)	2 (3.0)	0 (0.0)	0.478
	4A	38 (37.3)	23 (34.3)	15 (42.9)	
	4B	27 (26.5)	20 (29.9)	7 (20.0)	
	4C	32 (31.4)	21 (31.3)	11 (31.4)	
	5	3 (2.9)	1 (1.5)	2 (5.7)	
4B	0	75 (73.5)	47 (70.1)	28 (80.0)	0.349
	1	27 (26.5)	20 (29.9)	7 (20.0)	
4C	0	70 (68.6)	46 (68.7)	24 (68.6)	1
	1	32 (31.4)	21 (31.3)	11 (31.4)	
5	0	99 (97.1)	66 (98.5)	33 (94.3)	0.27
	1	3 (2.9)	1 (1.5)	2 (5.7)	
Enhanced model					0.304
0	No-enhancement	1 (1.0)	0 (0.0)	1 (2.9)	
1	Hetro,hypoenhancement	5 (4.9)	3 (4.5)	2 (5.7)	
2	Homo,hypoenhancement	9 (8.8)	6 (9.0)	3 (8.6)	
3	Hetero,hyperenhancement	60 (58.8)	40 (59.7)	20 (57.1)	
4	Homo,hyperenhancement	25 (24.5)	18 (26.9)	7 (20.0)	
**5**	isoenhancement	2 (2.0)	0 (0.0)	2 (5.7)	
**Model 3**	0	42 (41.2)	27 (40.3)	15 (42.9)	0.835
	1	60 (58.8)	40 (59.7)	20 (57.1)	
Model 4	0	77 (75.5)	49 (73.1)	28 (80.0)	0.48
	1	25 (24.5)	18 (26.9)	7 (20.0)	
Model 5	0	100 (98.0)	67 (100.0)	33 (94.3)	0.116
	1	2 (2.0)	0 (0.0)	2 (5.7)	
Model 4/5	0	75 (73.5)	49 (73.1)	26 (74.3)	1
	1	27 (26.5)	18 (26.9)	9 (25.7)	
The enhancement size	Equal	33 (32.4)	25 (37.3)	8 (22.9)	0.182
	Enlarged	69 (67.6)	42 (62.7)	27 (77.1)	
Border	Well-defined	83 (81.4)	54 (80.6)	29 (82.9)	1
	Ill-defined	19 (18.6)	13 (19.4)	6 (17.1)	
Shape	Regular	12 (11.8)	10 (14.9)	2 (5.7)	0.211
	Irregular	90 (88.2)	57 (85.1)	33 (94.3)	
Wash-in	Obsent	22 (21.6)	13 (19.4)	9 (25.7)	0.46
	Present	80 (78.4)	54 (80.6)	26 (74.3)	
Wash-out	Obsent	100 (98.0)	65 (97.0)	35 (100.0)	0.545
	Present	2 (2.0)	2 (3.0)	0 (0.0)	
Crab-like sign	Obsent	53 (52.0)	37 (55.2)	16 (45.7)	0.408
	Present	49 (48.0)	30 (44.8)	19 (54.3)	

### Nomogram model establishment

**Figure 1:**
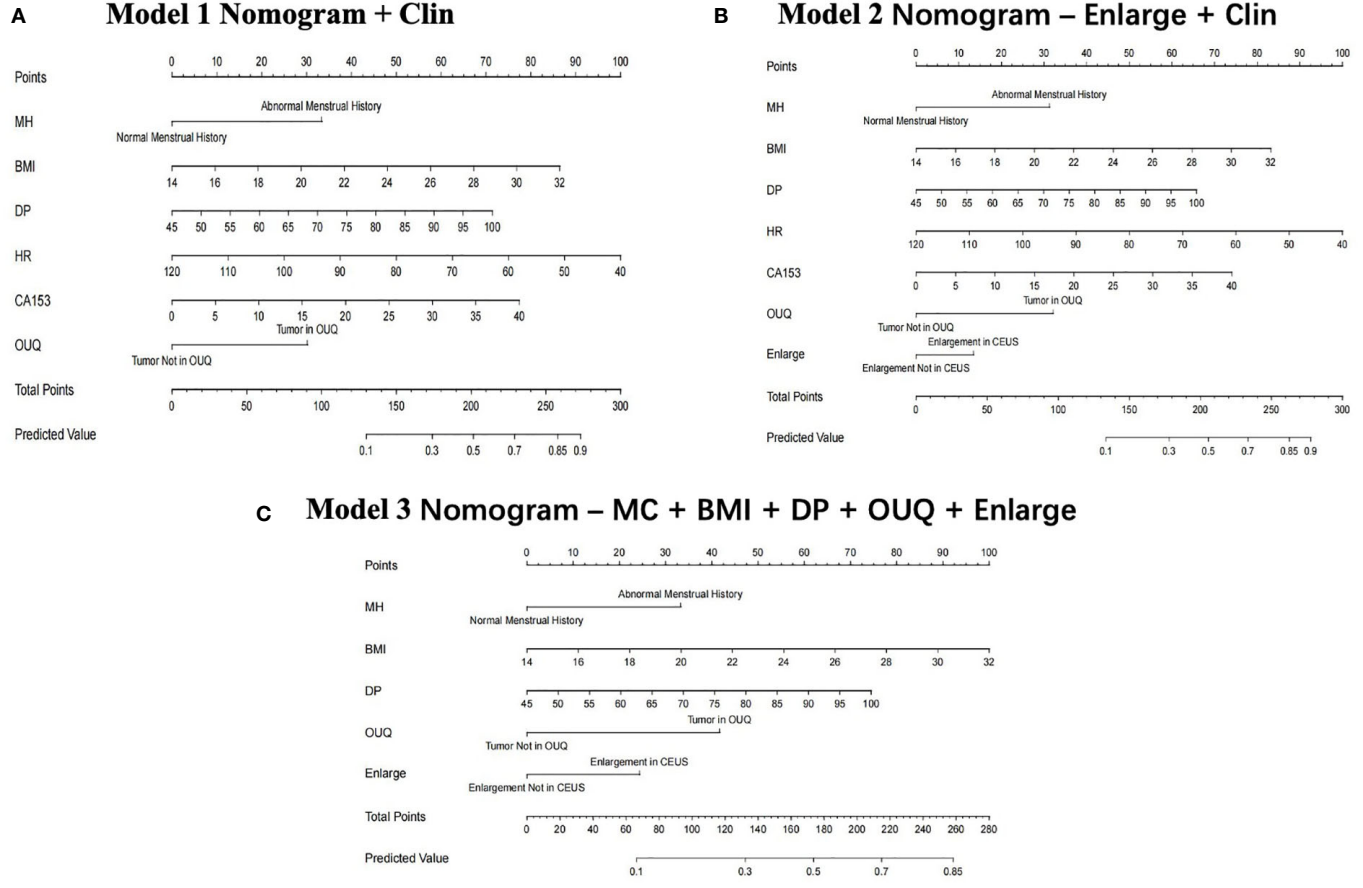
Clin, clinical features; MC, menstrual cycle; BMI, body mass index; DP, diastolic pressure; OUQ, outer upper quarter. Nomogram graphics of Clin model **(A)**, Enlarge + Clin model **(B)** and MC+BMI+ DP+ OUQ+ Enlarge model **(C)** for predicting Her-2 over-expression patients.

In the multivariate regression model I, OUQ was found to be the best predictor for Her-2 overexpressing with an odds ratio (OR) value of 2.52. For Model II, OUQ (OR=2.66) and an enlarged CEUS pattern (OR=1.51) were significant factors for predicting Her-2-overexpressing (**Table 2**). All nomogram figures are shown in **Figure 1**.

**Table 2:** Multivariate analysis of clinical and CEUS features for predicting Her-2 overexpressing in training cohort.

	OR (95% CI)	β value	*P* value
Clin			
MC	0.36(0.12,0.99)	-1.02	0.052
BMI	1.16(1.00,1.36)	0.15	0.053
DP	1.04(0.99,1.09)	0.04	0.099
HR	0.96(0.93,1.00)	-0.04	0.041
CA153	1.06(0.98,1.15)	0.06	0.139
OUQ	2.52(1.02,6.48)	0.93	0.048
Clin+Enlarge			
MC	0.39(0.13,1.08)	-0.95	0.074
BMI	1.15(0.99,1.35)	0.14	0.067
DP	1.04(0.99,1.09)	0.04	0.138
HR	0.96(0.93,1.00)	-0.04	0.044
CA153	1.06(0.98,1.15)	0.06	0.165
OUQ	2.66(1.06,6.99)	0.98	0.040
Enlarge	1.51(0.53,4.57)	0.41	0.453
MC+BMI+DP+OUQ+Enlarge			
MC	0.51(0.19,1.30)	-0.68	0.157
BMI	1.12(0.98,1.29)	0.11	0.099
DP	1.03(0.98,1.08)	0.03	0.228
OUQ	2.35(0.98,5.79)	0.85	0.058
Enlarge	1.65(0.62,4.69)	0.50	0.330

Clin, clinical features; MC, menstrual cycle; BMI, body mass index; DP, diastolic pressure; HR, heart rate; OUQ, outer upper quarter.

### Diagnostic performance of different models

**Figure 2:**
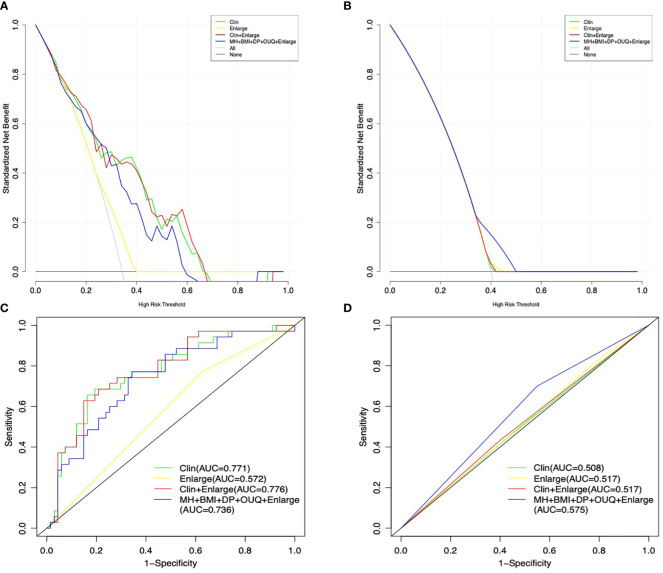
Decision curve graphics of all models for predicting Her-2 over-expression patients in the training **(A)** and test cohort **(B)**. AUC graphics of all models for predicting Her-2 positive patients in the training **(C)** and test cohort **(D)**.

Model II achieved an AUC value of 0.776 at nomogram cutoff score value of 190, higher than that of the other models in the training cohort, but without significant differences. In the test cohort, model II achieved a higher AUC value when compared to that of model I without significant differences (P=0.94) (**Table 3** and **Figure 2**).

**Table 3:** Diagnostic performance of different models for predicting Her-2 overexpressing in training and test cohort.

Model		Cut-off value	AUC	SEN	SPE	PPV	NPV
1	Clin	185					
	Training cohort		0.771	0.657	0.836	0.676	0.824
	Test cohort		0.472	0.75	0.367	0.441	0.688
2	Clin+Enlarge	190					
	Training cohort		0.776	0.629	0.851	0.688	0.814
	Test cohort		0.458	0.7	0.4	0.438	0.667
3	MC+BMI+DP+OUQ+Enlarge	138					
	Training cohort		0.736	0.771	0.657	0.54	0.846
	Test cohort		0.43	0.9	0.2	0.429	0.75

Clin, clinical features; MC, menstrual cycle; BMI, body mass index; DP, diastolic pressure; HR, heart rate; OUQ, outer upper quarter; AUC, area under the receiver operating characteristic; SEN, sensitivity; SPE, specificity; PPV, positive predictive values; NPV, negative predictive values.

In addition, the sensitivity and specificity were not significantly different between Models I and II (all *P*>0.05), in either the training or the test cohort. Moreover, model III exhibited a higher sensitivity (70.0%) than those of other models with similar AUC and specificity, only in the test cohort.

### Inter-reader agreement

The results showed that interobserver agreement on CEUS BI- RADS category was good.

## Discussion

The application of nomograms combined with multiple imaging modalities and clinical information is becoming increasingly popular in breast cancer research. It is not only used to identify malignancies and differentiate tumor grades, but also used to predict prognostic factors (23–25). Many studies have shown that nomograms can accurately discriminate breast cancer (23–26). The prognosis of Her-2-overexpressing cancers may be worse, however, patients with Her-2 overexpressing cancers could be effectively treated by targeted therapies, which are personalized and effective treatments (27, 28). The identification of positive Her-2 expression from the features extracted from medical information is an important issue in the clinical decision-making of breast cancer. In this study, we proposed a novel nomogram based on clinical, pathologic and CEUS features to predict Her-2 overexpression in OMs. The nomogram incorporated 9 possible predictors including MC, BMI, DP, HR, CA153 level, tumor size (maximum diameter), location (areola, OUQ), and enlargement. Our results showed the nomogram did not have good discrimination ability in either the training dataset or the validation dataset.

This study found that the clinical model contained several cardiovascular-related variables including BMI, DP, and HR. Several studies have proven that patients with breast cancer, especially those specific demographic characteristics, an elevated risk of developing cardiovascular diseases including hypertension heart failure, ischemic heart disease, cerebrovascular disease, peripheral vascular disease, and atrial fibrillation (29–31). However, neither the previous studies nor our study found a relationship among clinical features, cardiovascular diseases and the classification of breast cancer. Many factors are associated with cardiovascular disease, including age, lifestyle, metabolism, genetics, BMI, ovarian function, and emotional health. With large database studies including breast cancer subgroup classification, we hope that the relationship between breast cancer and cardiovascular disease may be explained more clearly.

For many years, tumor size has been used to evaluate the prognosis and determining the appropriate treatment strategy (32–34). In the present study, tumor sizes larger than 20 mm were found to be predictors for predicting Her-2 overexpression, but this correlation was not significant (OR=2.05, *P*=0.12) in the univariate regression analysis, which agrees with previous results (35). Invasive growth is one of the typical characteristics of all breast cancers, and size did not significantly different types. Similarly, Her-2 positivity was more frequently found in the OUQ than in other quadrants in this study (OR=2.24, *P*=0.06), which was consistent with past findings. Breast cancer is thought to most likely to occur in the OUQ, which is the quadrant with the highest breast area and a dense area (36). However, the incorporation of these features in a combined Clin model resulted in a poor diagnostic performance in predicting Her-2-overexpression (AUC=0.47, sensitivity=75.0%, and specificity=36.7%) in the test cohort. The proportion of dense breasts in Chinese women may be a contributing factor. Future study with the factor of breasts density is needed.

Her-2 overexpression increases vascular endothelial growth factor (VEGF) synthesis, which could increase angiogenesis in breast cancer. Angiogenesis, which differs among the various molecular types of breast cancer, is essential to the growth and metastasis of breast tumors since they are vascular-dependent. Clinical research, including our own, has concluded that hyper-enhancement, enlargement and crab-like sign on the CEUS pattern is more common in malignant breast lesions than in benign lesions (20–22). Previous study showed that Her-2 over-expression subtype contrast enhancement pattern was more frequently present with centripetal enhancement with a perfusion defect (37). However, no currently existing study has yet reported the effectiveness of the combination of clinical information and CEUS features in predicting breast cancer subtypes. In the era of personalized or precision medicine, the integration of nomograms based on clinical, and CEUS features may increase the possibility for clinicians to plan patient-centered treatments. The Her-2 over-expression subtype expresses VEGF in high levels, which can stimulate tumor angiogenesis from the tumor’s periphery to its core. As a result, the heterogeneity of VEGF expression within the tumor is greater than that of the tumor mass itself, and this distinguishes the Her-2 over-expression subtype’s contrast enhancement pattern from that of other cancers. Unfortunately, in the current study, the combination of clinical and CEUS features showed no significant diagnostic performance in predicting Her-2-overexpression compared with the clinical model alone. We also established a model based on several types of clinical information that could enhance the efficacy of the “enlargement” sign. However, the result was not satisfactory. This lack of effect may be affected by factors such as the size of the included lesions and the sample size. Also, the quantitative analysis of CEUS was not included in the study. In this respect, further studies from larger trials will be necessary to achieve prediction of Her-2 through preoperative information.

This study had some limitations. First, the retrospective nature of the training set may have led to unavoidable selection bias. Therefore, a prospective study is required to achieve the predictive model. Second, the sample size was relatively small, especially in the test cohort. Third, different US machines were used to collect CEUS data at the two centers, which may result in image variability. The limited number of Her-2-overexpressing breast cancer patients in the test cohort inhibits subgroup evaluation of US-machine-derived inconsistencies. This problem could be solved by conducting a prospective multicenter study of a large sample. Finally, a quantitative analysis of interpreting CEUS features would be much better and required to address the inconsistency associated with naked-eye observation. Further research will need to be conducted in the future.

## Conclusions

In summary, the nomogram is based on clinical and CEUS features that can be obtained in a preoperative setting. The proposed nomogram could not be used to individually predict Her-2-overexpression in breast cancer patients. The results may indicate the need for a deep study to obtain more meaningful results for clinical application.

## Data availability statement

The raw data supporting the conclusions of this article will be made available by the authors, without undue reservation.

## Ethics statement

The studies involving human participants were reviewed and approved by The Second Affiliated Hospital Zhejiang University School of Medicine and The First Affiliated Hospital of Zhejiang Chinese Medical University. The patients/participants provided their written informed consent to participate in this study.

## Author contributions

All authors contributed to the conception and design of the study. The entire study was chaired by PTH. ZML and TTW contributed significantly to the analysis and manuscript preparation and wrote the manuscript, and they contributed equally to this study. ZML, TTW, JYZ, YYX helped perform the analysis with constructive discussions. All authors contributed to the article and approved the submitted version.
