# Supplementary material for: A nomogram based on combining clinical features and contrast enhanced ultrasound is not able to identify Her-2 over-expressing cancer from other breast cancers
Source: Front Oncol. 2023 Jan 26;13:1035645. doi: 10.3389/fonc.2023.1035645 (PMC9909531; doi:10.3389/fonc.2023.1035645)
Supplement: Supplementary file 1 [file DataSheet_1.doc]

**Supplemental information**

**Supplemental Table 1. Basic information between training cohort and test cohort**

| (0=no;1=yes) (%) | Level | Training Cohort (n=102) (%) | Test Cohort (n=50) (%) | P value |
| --- | --- | --- | --- | --- |
| Age (mean (SD)) | | 55.96 (12.57) | 55.64 (9.77) | 0.874 |
| Menstruation | 0 | 31 (30.4) | 13 (26.0) | 0.711 |
|  | 1 | 71 (69.6) | 37 (74.0) |  |
| BMI (median [IQR]) | | 22.70 [21.23, 24.99] | 23.23 [21.84, 24.22] | 0.718 |
| BMI M25 | 0 | 78 (76.5) | 42 (84.0) | 0.391 |
|  | 1 | 24 (23.5) | 8 (16.0) |  |
| Systolic (mean (SD)) | | 123.65 (15.78) | 127.46 (15.19) | 0.159 |
| Systolic | 0 | 89 (87.3) | 42 (84.0) | 0.767 |
|  | 1 | 13 (12.7) | 8 (16.0) |  |
| Diastolic (mean (SD)) | | 75.53 (9.98) | 75.90 (10.38) | 0.83 |
| Diastolic M90 | 0 | 94 (92.2) | 46 (92.0) | 1 |
|  | 1 | 8 (7.8) | 4 (8.0) |  |
| Diastolic M80 | 0 | 69 (67.6) | 35 (70.0) | 0.914 |
|  | 1 | 33 (32.4) | 15 (30.0) |  |
| Heartrate (mean (SD)) | | 81.24 (14.27) | 80.52 (10.64) | 0.751 |
| Heartrate M100 | 0 | 93 (91.2) | 48 (96.0) | 0.456 |
|  | 1 | 9 (8.8) | 2 (4.0) |  |
| CA153 (median [IQR]) | | 9.00 [6.40, 11.78] | 8.90 [6.30, 10.93] | 0.606 |
| CA153 M14 | 0 | 87 (85.3) | 46 (92.0) | 0.361 |
|  | 1 | 15 (14.7) | 4 (8.0) |  |
| CA153 M20 | 0 | 96 (94.1) | 50 (100.0) | 0.191 |
|  | 1 | 6 (5.9) | 0 (0.0) |  |
| CA153 M25 | 0 | 98 (96.1) | 50 (100.0) | 0.379 |
|  | 1 | 4 (3.9) | 0 (0.0) |  |
| Lesion location (1=left;2=right) | 1 | 57 (55.9) | 32 (64.0) | 0.436 |
|  | 2 | 45 (44.1) | 18 (36.0) |  |
| Lesion o'clock (0=areola) | 0 | 19 (18.6) | 8 (16.0) | 0.247 |
|  | 1 | 6 (5.9) | 2 (4.0) |  |
|  | 2 | 16 (15.7) | 10 (20.0) |  |
|  | 3 | 7 (6.9) | 5 (10.0) |  |
|  | 4 | 6 (5.9) | 0 (0.0) |  |
|  | 5 | 3 (2.9) | 0 (0.0) |  |
|  | 6 | 0 (0.0) | 1 (2.0) |  |
|  | 7 | 7 (6.9) | 0 (0.0) |  |
|  | 8 | 4 (3.9) | 4 (8.0) |  |
|  | 9 | 4 (3.9) | 5 (10.0) |  |
|  | 10 | 15 (14.7) | 9 (18.0) |  |
|  | 11 | 4 (3.9) | 3 (6.0) |  |
|  | 12 | 11 (10.8) | 3 (6.0) |  |
| Areola | 0 | 83 (81.4) | 42 (84.0) | 0.863 |
|  | 1 | 19 (18.6) | 8 (16.0) |  |
| OUQ | 0 | 57 (55.9) | 21 (42.0) | 0.151 |
|  | 1 | 45 (44.1) | 29 (58.0) |  |
| Size (median [IQR]) | | 1.54 [1.13, 2.06] | 1.79 [1.43, 2.85] | 0.024 |
| Size M2 | 0 | 74 (72.5) | 28 (56.0) | 0.063 |
|  | 1 | 28 (27.5) | 22 (44.0) |  |
| Size M2.5 | 0 | 86 (84.3) | 34 (68.0) | 0.035 |
|  | 1 | 16 (15.7) | 16 (32.0) |  |
| Size M3 | 0 | 93 (91.2) | 40 (80.0) | 0.09 |
|  | 1 | 9 (8.8) | 10 (20.0) |  |
| BIRADS category | 3 | 0 (0.0) | 1 (2.0) | 0.535 |
|  | 4A | 37 (36.3) | 19 (38.0) |  |
|  | 4B | 29 (28.4) | 13 (26.0) |  |
|  | 4C | 33 (32.4) | 14 (28.0) |  |
|  | 5 | 3 (2.9) | 3 (6.0) |  |
| BIRADS 4B | 0 | 73 (71.6) | 37 (74.0) | 0.903 |
|  | 1 | 29 (28.4) | 13 (26.0) |  |
| BIRADS 4C | 0 | 69 (67.6) | 36 (72.0) | 0.72 |
|  | 1 | 33 (32.4) | 14 (28.0) |  |
| BIRADS 5 | 0 | 99 (97.1) | 47 (94.0) | 0.641 |
|  | 1 | 3 (2.9) | 3 (6.0) |  |

Supplemental Table 2. Univariate analysis of clinical, US and CEUS features for predicting MVI status in test cohort

|  | Level | Overall | OMs | Her-2+ | *P* |
| --- | --- | --- | --- | --- | --- |
| n |  | 50 | 30 | 20 |  |
| age (mean (SD)) | | 55.96 (12.57) | 55.64 (9.77) | 57.40 (9.30) | 53.00 (10.09) |
| Menstruation(0=no;1=yes) | 0 | 13 (26.0) | 6 (20.0) | 7 (35.0) | 0.327 |
|  | 1 | 37 (74.0) | 24 (80.0) | 13 (65.0) |  |
| BMI (median [IQR]) | | 22.70 [21.23, 24.99] | 23.14 (1.89) | 22.76 (1.91) | 23.71 (1.75) |
| BMI M25 | 0 | 42 (84.0) | 26 (86.7) | 16 (80.0) | 0.697 |
|  | 1 | 8 (16.0) | 4 (13.3) | 4 (20.0) |  |
| Systolic (mean (SD)) | | 123.65 (15.78) | 125.50 [117.00, 138.50] | 133.00 [117.50, 140.00] | 118.00 [113.00, 126.00] |
| Systolic M140 | 0 | 42 (84.0) | 24 (80.0) | 18 (90.0) | 0.45 |
|  | 1 | 8 (16.0) | 6 (20.0) | 2 (10.0) |  |
| Diastolic (mean (SD)) | | 75.53 (9.98) | 75.90 (10.38) | 77.37 (10.81) | 73.70 (9.55) |
| Diastolic M90 | 0 | 46 (92.0) | 26 (86.7) | 20 (100.0) | 0.14 |
|  | 1 | 4 (8.0) | 4 (13.3) | 0 (0.0) |  |
| Diastolic M80 | 0 | 35 (70.0) | 19 (63.3) | 16 (80.0) | 0.345 |
|  | 1 | 15 (30.0) | 11 (36.7) | 4 (20.0) |  |
| Heart rate (mean (SD)) | | 81.24 (14.27) | 80.00 [76.00, 85.75] | 80.50 [70.50, 83.00] | 79.50 [76.75, 87.25] |
| Heartrate M100 | 0 | 48 (96.0) | 30 (100.0) | 18 (90.0) | 0.155 |
|  | 1 | 2 (4.0) | 0 (0.0) | 2 (10.0) |  |
| CA153 (median [IQR]) | | 9.00 [6.40, 11.78] | 8.97 (3.25) | 8.77 (3.16) | 9.27 (3.44) |
| CA153 M14 | 0 | 46 (92.0) | 27 (90.0) | 19 (95.0) | 0.641 |
|  | 1 | 4 (8.0) | 3 (10.0) | 1 (5.0) |  |
| CA153 M20 | 0 | 50 (100.0) | 30 (100.0) | 20 (100.0) | NA |
|  | 1 | 50 (100.0) | 30 (100.0) | 20 (100.0) | NA |
| CA153 M25 | 0 | 32 (64.0) | 18 (60.0) | 14 (70.0) | 0.556 |
|  | 1 | 18 (36.0) | 12 (40.0) | 6 (30.0) |  |
| Lesion location(1=left;2=right) | 1 | 8 (16.0) | 5 (16.7) | 3 (15.0) | 0.341 |
|  | 2 | 2 (4.0) | 2 (6.7) | 0 (0.0) |  |
| o'clock (0=areola;13=chestwall) | 0 | 10 (20.0) | 7 (23.3) | 3 (15.0) |  |
|  | 1 | 5 (10.0) | 2 (6.7) | 3 (15.0) |  |
|  | 2 | 1 (2.0) | 1 (3.3) | 0 (0.0) |  |
|  | 3 | 4 (8.0) | 1 (3.3) | 3 (15.0) |  |
|  | 4 | 5 (10.0) | 4 (13.3) | 1 (5.0) |  |
|  | 5 | 9 (18.0) | 3 (10.0) | 6 (30.0) |  |
|  | 7 | 3 (6.0) | 3 (10.0) | 0 (0.0) |  |
|  | 8 | 3 (6.0) | 2 (6.7) | 1 (5.0) |  |
|  | 9 | 42 (84.0) | 25 (83.3) | 17 (85.0) | 1 |
|  | 10 | 8 (16.0) | 5 (16.7) | 3 (15.0) |  |
|  | 11 | 21 (42.0) | 11 (36.7) | 10 (50.0) | 0.393 |
|  | 12 | 29 (58.0) | 19 (63.3) | 10 (50.0) |  |
| Areola | 0 | 1.79 [1.43, 2.85] | 1.71 [1.16, 2.52] | 1.80 [1.68, 3.02] | 0.118 |
|  | 1 | 28 (56.0) | 17 (56.7) | 11 (55.0) | 1 |
| OUQ | 0 | 22 (44.0) | 13 (43.3) | 9 (45.0) |  |
|  | 1 | 34 (68.0) | 22 (73.3) | 12 (60.0) | 0.366 |
| Size (median [IQR]) | | 1.54 [1.13, 2.06] | 16 (32.0) | 8 (26.7) | 8 (40.0) |
| Size M2 | 0 | 40 (80.0) | 26 (86.7) | 14 (70.0) | 0.171 |
|  | 1 | 10 (20.0) | 4 (13.3) | 6 (30.0) |  |
| Size M2.5 | 0 | 1 (2.0) | 0 (0.0) | 1 (5.0) | 0.55 |
|  | 1 | 19 (38.0) | 13 (43.3) | 6 (30.0) |  |
| Size M3 | 0 | 13 (26.0) | 6 (20.0) | 7 (35.0) |  |
|  | 1 | 14 (28.0) | 9 (30.0) | 5 (25.0) |  |
| BIRADS category | 3 | 3 (6.0) | 2 (6.7) | 1 (5.0) |  |
|  | 4A | 37 (74.0) | 24 (80.0) | 13 (65.0) | 0.327 |
|  | 4B | 13 (26.0) | 6 (20.0) | 7 (35.0) |  |
|  | 4C | 36 (72.0) | 21 (70.0) | 15 (75.0) | 0.758 |
| BIRADS 4B | 0 | 14 (28.0) | 9 (30.0) | 5 (25.0) |  |
|  | 1 | 47 (94.0) | 28 (93.3) | 19 (95.0) | 1 |
| BIRADS 4C | 0 | 3 (6.0) | 2 (6.7) | 1 (5.0) |  |
|  | 1 | 19 (38.0) | 11 (36.7) | 8 (40.0) | 0.832 |
| BIRADS 5 | 0 | 26 (52.0) | 15 (50.0) | 11 (55.0) |  |
|  | 1 | 5 (10.0) | 4 (13.3) | 1 (5.0) |  |
| Elastography | 2 | 31 (62.0) | 19 (63.3) | 12 (60.0) | 1 |
|  | 3 | 19 (38.0) | 11 (36.7) | 8 (40.0) |  |
|  | 4 | 24 (48.0) | 15 (50.0) | 9 (45.0) | 0.779 |
|  | 5 | 26 (52.0) | 15 (50.0) | 11 (55.0) |  |
| E3 | 0 | 45 (90.0) | 26 (86.7) | 19 (95.0) | 0.636 |
|  | 1 | 5 (10.0) | 4 (13.3) | 1 (5.0) |  |
| E4 | 0 | 1 (2.0) | 1 (3.3) | 0 (0.0) | 0.948 |
|  | 1 | 20 (40.0) | 12 (40.0) | 8 (40.0) |  |
| E5 | 0 | 12 (24.0) | 6 (20.0) | 6 (30.0) |  |
|  | 1 | 14 (28.0) | 9 (30.0) | 5 (25.0) |  |
| CEUS BIRADS category | 3 | 3 (6.0) | 2 (6.7) | 1 (5.0) |  |
|  | 4A | 38 (76.0) | 24 (80.0) | 14 (70.0) | 0.506 |
|  | 4B | 12 (24.0) | 6 (20.0) | 6 (30.0) |  |
|  | 4C | 36 (72.0) | 21 (70.0) | 15 (75.0) | 0.758 |
|  | 5 | 14 (28.0) | 9 (30.0) | 5 (25.0) |  |
| 4B | 0 | 47 (94.0) | 28 (93.3) | 19 (95.0) | 1 |
|  | 1 | 3 (6.0) | 2 (6.7) | 1 (5.0) |  |
| 4C | 0 | 6 (12.0) | 3 (10.0) | 3 (15.0) | 0.689 |
|  | 1 | 4 (8.0) | 2 (6.7) | 2 (10.0) |  |
| 5 | 0 | 28 (56.0) | 16 (53.3) | 12 (60.0) |  |
|  | 1 | 12 (24.0) | 9 (30.0) | 3 (15.0) |  |
| Enhanced model |  |  |  |  |  |
| 0 | No-enhancement | 22 (44.0) | 14 (46.7) | 8 (40.0) | 0.773 |
| 1 | Hetro,hypoenhancement | 28 (56.0) | 16 (53.3) | 12 (60.0) |  |
| 2 | Homo,hypoenhancement | 38 (76.0) | 21 (70.0) | 17 (85.0) | 0.317 |
| 3 | Hetero,hyperenhancement | 12 (24.0) | 9 (30.0) | 3 (15.0) |  |
| 4 | Homo,hyperenhancement | 50 (100.0) | 30 (100.0) | 20 (100.0) | NA |
| 5 | isoenhancement | 38 (76.0) | 21 (70.0) | 17 (85.0) | 0.317 |
| Model 3 | 0 | 12 (24.0) | 9 (30.0) | 3 (15.0) |  |
|  | 1 | 9 (18.0) | 5 (16.7) | 4 (20.0) | 1 |
| Model 4 | 0 | 41 (82.0) | 25 (83.3) | 16 (80.0) |  |
|  | 1 | 49 (98.0) | 30 (100.0) | 19 (95.0) | 0.4 |
| Model 5 | 0 | 1 (2.0) | 0 (0.0) | 1 (5.0) |  |
|  | 1 | 1 (2.0) | 0 (0.0) | 1 (5.0) | 0.4 |
| Model 4/5 | 0 | 49 (98.0) | 30 (100.0) | 19 (95.0) |  |
|  | 1 | 2 (4.0) | 1 (3.3) | 1 (5.0) | 1 |
| The enhancement size | Equal | 48 (96.0) | 29 (96.7) | 19 (95.0) |  |
|  | Enlarged | 48 (96.0) | 29 (96.7) | 19 (95.0) | 1 |
| Border | Well-defined | 2 (4.0) | 1 (3.3) | 1 (5.0) |  |
|  | Ill-defined | 13 (26.0) | 7 (23.3) | 6 (30.0) | 0.744 |
| Shape | Regular | 37 (74.0) | 23 (76.7) | 14 (70.0) |  |
|  | Irregular | 50 | 30 | 20 |  |
| Wash-in | Obsent | 55.64 (9.77) | 57.40 (9.30) | 53.00 (10.09) | 0.12 |
|  | Present | 13 (26.0) | 6 (20.0) | 7 (35.0) | 0.327 |
| Wash-out | Obsent | 37 (74.0) | 24 (80.0) | 13 (65.0) |  |
|  | Present | 23.14 (1.89) | 22.76 (1.91) | 23.71 (1.75) | 0.083 |
| Sun-sign | Obsent | 42 (84.0) | 26 (86.7) | 16 (80.0) | 0.697 |
|  | Present | 8 (16.0) | 4 (13.3) | 4 (20.0) |  |
